# Health Tract, No. 290

**Published:** 1867-05

**Authors:** 


HEALTH TRACT, No. 290.

### WOMAN.

Angels are the only unabused beings in the universe, but woman, who is only a mite lower than the angels, is the most abused! by that class of men who are vulgar in their natures, brutal in their passions and degraded in their whole being. When we hear a man speak disparagingly of women, as a class; he that very instant becomes so degraded in our estimation, that a lifetime of faultless conduct could not elevate him in our eyes, to the standard of a gentleman. Who but the lowest grovellers can think of mother, sister, wife, daughter, with other than feelings of respect and affection and love. And yet some crazy poet has written,

> "O woman, woman! thou art like
> To Jeremiah's figs,
> The good are very good indeed,
> The bad too sour for pigs."

But even this fellow, scape grace as he must have been, could not have for a moment thought his mother, wife, sister or daughter, anything else than "very good," in the sense that all His works are, who made the stars, the sunshine and the flowers.

Celebrities have said several things of woman which, while they are suggestive, challenge indignant contradiction! The very last words of an unfortunate under the gallows at the New York Tombs, in 1854, were, "I didn't intend to kill my wife, but she was a werry aggrawating woman." Now reader, I put it to you, in real earnest and "'pon honor," did you ever in all your life think for a moment, that the dear creature who hangs so lovingly on your arm as when, like a good man you go to church on the Sabbath day, was aggravating? Certainly you can't remember with specific definiteness the circumstances, the time when, and the place where such an unwelcome intruder forced itself on your attention. Harriet Beecher Stowe, who is both man and wife, for we never heard anything of Mr. Stowe; certainly never hung up anybody—except Uncle Tom to the gaze of the world, quotes with seeming approval the great Balzac as saying, "Woman is incapable of logic;" and a very staid placid Quaker friend of ours, who lives in grand style on the retiracy of his own made ample fortune; and said it too with a vim, rather remarkable for a drab, "women have no regard for consequences." And a very cavalier said several years ago, his beautiful and accomplished and most devoted wife standing by his side, in our office, and the sentiment was as new as it was startling. A high legal authority in England, has very ungallantly said, "I don't know how it is, but somehow or other, women are constitutionally incapable of speaking the truth."

Reader, when you see such ideas as these put on paper, regard them as "the baseless fabric of a vision," and as a means of verification, follow the advice of a physician who has seen sights! "As soon as practicable take, not Dr. Quack's celebrated cordial and elixir, but if you have never done it before, take a woman, make her your wife, and you will be a better, happier and a ———————— wiser man; and so promotive of health is this wife taking, that in all climes and countries, bachelors die earlier, on an average, by several years, than the married."

